# Tai Chi, brain activity and psychological outcomes: a systematic scoping review

**DOI:** 10.3389/fpsyg.2026.1865028

**Published:** 2026-06-04

**Authors:** Fengshan Yue, Nan Chen, Hao He, Xiaolei Zhang, Cheng Pan, He Yang

**Affiliations:** 1 Department of Physical Education, Northwestern Polytechnical University, Xi’an, Shaanxi, China; 2 Health and Physical Education Department of Xi'An Fanyi University, Xi’an, Shaanxi, China; 3 Institute of Physical Education, Northeast Normal University, Changchun, China; 4 Guangzhou Applied Science University Sports Science College, Zhaoqing, China; 5 School of Physical Education, Hanjiang Normal University, Shiyan, China; 6 Public Teaching Department, Xinyang Aviation Vocational College, Xinyang, China

**Keywords:** brain activity, neuroimaging, psychological outcomes, scoping review, Tai Chi

## Abstract

**Objectives:**

To identify and map studies examining Tai Chi in relation to at least one measure of brain activity or brain function and at least one psychological outcome; to characterize the populations, Tai Chi exposure features, neural modalities, and psychological domains represented; and to summarize whether included studies reported temporal or statistical links between neural and psychological findings.

**Methods:**

PubMed, Scopus, and Web of Science Core Collection were searched without date restrictions. Eligible studies reported original human data on Tai Chi and included at least one functional or neurophysiological brain outcome and at least one psychological or closely related cognitive-emotional outcome. Records were screened, full texts assessed, data extracted, and design-specific critical appraisal conducted. Findings were synthesized descriptively using evidence tables, narrative synthesis, and an evidence gap map.

**Results:**

Of 838 records identified, 460 unique records were screened after deduplication, 25 full texts were assessed, and 22 studies were included. The evidence base comprised randomized, non-randomized, and cross-sectional comparative studies. Older adults and university students were the most frequent populations, although several clinical groups were also represented. Electroencephalography and event-related potentials, functional magnetic resonance imaging, and functional near-infrared spectroscopy were the principal neural modalities. Across modalities, the most consistent pattern was the co-occurrence of favorable psychological or cognitive-emotional findings with Tai Chi-related neural change, although only a subset of studies formally tested eligible brain-behavior associations. In an added association-direction synthesis, statistically significant favorable associations were most evident in functional magnetic resonance imaging studies of depression, fatigue, and emotion-related outcomes. Favorable associations were also observed in electroencephalography/event-related-potential studies of anxiety and cognitive-emotional outcomes, and in one functional near-infrared spectroscopy study of emotional memory. However, many studies reported parallel neural and psychological findings without a direct eligible association test.

**Conclusion:**

This literature is growing but remains methodologically heterogeneous and unevenly distributed. It identifies important evidence clusters and gaps, but does not yet support specific mechanisms that support conclusions regarding effectiveness. The findings support cautious consideration of Tai Chi as a low-intensity adjunctive mind–body practice for psychological and cognitive-emotional outcomes, while indicating that neural measures should currently be used to improve trial design and hypotheses rather than to guide routine clinical decision-making.

**Systematic review registration:**

https://osf.io/fup6c/overview, Open Science Framework (ID: osf.io/fup6c; Date: 17-04-2026).

## 1 Introduction

Tai Chi is a traditional Chinese mind–body practice characterized by coordinated movement, postural control, breathing regulation, and focused attention (Larkey et al., 2009; Yeung et al., 2018). Across clinical and nonclinical populations, Tai Chi has been studied as a non-pharmacological intervention for outcomes such as stress, anxiety, depression, mood disturbance, and broader psychological well-being (Wang et al., 2010, 2014). Recent quantitative syntheses also suggest that Tai Chi may improve anxiety, depressive symptoms, or perceived stress in at least some populations, although effect estimates vary according to population, comparator, intervention dose, and study quality (Kraft et al., 2024; Kuang et al., 2024; Zhu et al., 2024).

In the neuroscience literature, Tai Chi has been examined using functional and neurophysiological methods including functional magnetic resonance imaging, electroencephalography, functional near-infrared spectroscopy, and magnetic resonance spectroscopy to investigate brain activation, connectivity, oscillatory activity, cerebral hemodynamics, or brain metabolism (Pan et al., 2018; Cui et al., 2019; Chen et al., 2022; Wang and Lyu, 2024; Wang et al., 2025). Primary studies have reported Tai Chi-associated changes in resting-state or task-related neural measures in older adults, university students, and clinical samples, including altered default mode or insular connectivity, greater prefrontal activation, and changes in frontal EEG band power (Tao et al., 2017; Wu et al., 2018; Liu et al., 2019; Xu et al., 2020; Chen et al., 2022; Wang and Lyu, 2024). Several of these studies have concurrently measured psychological or related mental outcomes, including anxiety, depression, stress, fatigue, emotional memory, or psychoemotional state (Xu et al., 2020; Solianik et al., 2021; Zhang et al., 2023; Wang et al., 2025).

The literature on psychological outcomes has already been synthesized in some systematic reviews and meta-analyses, which have evaluated Tai Chi in relation to psychological well-being, depression, anxiety, and stress across heterogeneous populations (Wang et al., 2010, 2014; Sani et al., 2023). By contrast, neuroimaging-focused evidence synthesis has been comparatively limited. For instance a systematic review published in 2018 (Pan et al., 2018) identified only 11 eligible studies in healthy older adults and found that the field was dominated by fMRI and EEG designs. Since that review, the field has expanded to additional populations and modalities, including pilot resting-state fMRI studies in major depressive disorder and in college students with anxiety/depression, randomized or controlled EEG studies in university students, and fNIRS studies of prefrontal activation in older adults and during acute Tai Chi tasks (Xu et al., 2020; Zhang et al., 2023; Wang and Lyu, 2024; Wang et al., 2025).

Despite this growth, the evidence base remains methodologically heterogeneous with respect to population characteristics, Tai Chi style and regimen, comparator conditions, neural acquisition and analysis methods, and psychological outcome selection (Wang et al., 2014; Pan et al., 2018). Existing reviews have either synthesized psychological outcomes without integrating neural measures, or summarized brain imaging and EEG studies in older adults without specifically mapping concurrent psychological outcomes across populations and contexts (Wang et al., 2010, 2014; Pan et al., 2018). A scoping review is especially appropriate when the aim is to map the breadth, characteristics, and knowledge gaps of a heterogeneous body of evidence rather than to estimate a single pooled intervention effect (Tricco et al., 2018; Peters et al., 2020).

Against this background, a systematic scoping review is warranted to integrate the currently dispersed literature at the intersection of Tai Chi, brain activity, and psychological outcomes. The objectives of this review were to identify and map human studies that examined Tai Chi in relation to at least one brain-activity measure and at least one psychological outcome; to describe the populations, Tai Chi exposure characteristics, neural modalities, and psychological domains represented in the literature; and to summarize whether included studies reported temporal or statistical links between neural findings and psychological outcomes.

## 2 Methods

This review was designed as a systematic scoping review. The reporting structure was aligned with PRISMA 2020 and, because the objective was to map a heterogeneous evidence base rather than to estimate a pooled intervention effect, was also informed by the PRISMA extension for scoping reviews (PRISMA-ScR) (Tricco et al., 2018). Search reporting was prepared in line with core PRISMA-S principles (Rethlefsen et al., 2021). The review protocol was published *a priori* in the Open Science Framework (ID: osf.io/fup6c; 17-04-2026).

### 2.1 Review question and eligibility framework

The review question was structured using a Population-Concept-Context framework. The population was humans of any age, sex, and health status. The core concept was Tai Chi, either as a structured intervention, a routine practice exposure, or a comparator-relevant activity clearly identifiable as Tai Chi, together with the reporting of both (i) at least one eligible measure of brain activity or brain function and (ii) at least one eligible psychological outcome. Context was unrestricted and included community, educational, laboratory, rehabilitation, and clinical settings.

Studies were eligible when they reported original human data on Tai Chi and included at least one central nervous system functional, neurophysiological, hemodynamic, connectivity, or brain-metabolic outcome intended to index brain activity or brain function, such as EEG or event-related potentials, functional MRI or resting-state functional MRI, functional near-infrared spectroscopy, positron-emission tomography, or brain magnetic resonance spectroscopy. Purely structural neuroimaging studies without a functional or neurophysiological neural outcome were not considered eligible for this study because the review question was centered on brain activity rather than brain morphology.

Eligible psychological outcomes included validated self-reported or investigator-administered measures of depression, anxiety, stress, mood, affect, fatigue, psychological well-being, mental health, or closely related neuropsychological or cognitive-emotional outcomes when these were explicitly analyzed as mental or psychological endpoints within the study. Studies reporting only motor, balance, cardiorespiratory, biomechanical, or other non-psychological outcomes were excluded unless at least one eligible psychological outcome was also reported.

Randomized controlled trials, non-randomized intervention studies, pre-post studies, cohort studies, case–control studies, and cross-sectional studies were eligible if they contained original data. Reviews, meta-analyses, protocols, editorials, commentaries, letters without primary data, conference abstracts without sufficient full-text detail, dissertations not indexed as full articles, animal studies, and purely methodological papers were excluded. For multicomponent interventions, studies were eligible only when the Tai Chi component was clearly described and its data were separable at the study-arm or analysis level.

### 2.2 Information sources

The literature search was conducted on April 17, 2026, in PubMed, Scopus, and Web of Science Core Collection. No date restrictions were applied. The search was conceived to maximize retrieval sensitivity at the database stage. Therefore, no study-design filter was used. The reference lists of included articles and closely related reviews were also checked manually. This manual checking step was used to reduce the likelihood that relevant studies indexed outside the three electronic databases would be missed, although it could not fully substitute for searching additional disciplinary, regional, or grey-literature databases.

### 2.3 Search strategy

The electronic search strategy was developed iteratively in PubMed before being translated to Scopus and Web of Science. To improve term selection, an initial scoping exercise was performed by examining the indexing and title/abstract terminology used in relevant reviews and primary studies on Tai Chi, neuroimaging, EEG, fNIRS, and psychological outcomes. The search concepts were derived from the review question and focused on Tai Chi and brain-function terminology. Psychological terms informed screening and data-charting decisions but were not made mandatory in the core electronic strategy in order to preserve sensitivity for neuroimaging and neurophysiology studies that might not mention mental outcomes in the title or abstract despite reporting them in the full text.

The searches were run without date limits. No language restriction was applied at the search stage.

[Title/abstract] “Tai ji” OR “tai chi” OR taiji OR “tai ji” OR taijiquan OR “t’ai chi” OR taichi.

AND

[Title/abstract] Electroencephalography OR electroencephalograph* OR EEG OR ERP OR “event-related potential*” OR “Magnetic Resonance Imaging” OR “functional magnetic resonance imaging” OR fMRI OR rs-fMRI OR “functional connectivity” OR “default mode network” OR fALFF OR ALFF OR “brain network*” OR “brain activ*” OR “brain function*” OR “neural activ*” OR “cortical activ*” OR “prefrontal activ*” OR “brain connectivity” OR “resting state” OR “Near-Infrared Spectroscopy” OR fNIRS OR “near infrared spectroscopy” OR “Magnetic Resonance Spectroscopy” OR MRS OR “brain metabol*” OR “Positron-Emission Tomography” OR PET OR neuroimaging OR neurophysiolog* OR neuroelectric* OR “brain hemodynamic*” OR “cerebral hemodynamic*” OR oxyhemoglobin OR “oxygenated hemoglobin” OR deoxyhemoglobin OR “deoxygenated hemoglobin.”

### 2.4 Selection process

All records were exported into the EndNote management environment, and duplicate citations were removed before screening. Title and abstract screening was then performed independently by two authors against the prespecified eligibility criteria. The full texts of potentially relevant records were retrieved and assessed independently by the same two authors. Disagreements at either stage were resolved by discussion and, when necessary, adjudication by a third author. For reports excluded at full-text assessment, the primary reason for exclusion was assigned according to the prespecified eligibility domain that was not met and was summarized with counts in the PRISMA flow diagram.

### 2.5 Data collection process

A standardized data-charting form was developed *a priori* and refined iteratively after pilot use on a small sample of eligible studies. Data extraction was carried out independently by two authors. Any discrepancies were resolved by consensus, with arbitration by a third author. When reports from the same study population were identified, they were linked and handled as companion publications, with the most complete dataset retained as the primary source and supplementary information extracted from companion reports where relevant.

### 2.6 Data items

For each included study, the following information was charted: bibliographic details; country and setting; study design; sample size; participant age, sex, and health status; and inclusion of healthy versus clinical populations. We also extracted Tai Chi style, format, frequency and regimen, session duration, weekly frequency, total intervention length, comparator conditions, and whether the exposure was acute or longitudinal. Neural data items included brain-activity modality, acquisition context, and analytic metrics, such as functional connectivity, band power, hemodynamic response, network indices, or brain metabolites. Psychological data items included outcome domains, measurement instruments, assessment time points, key findings, and any reported association, mediation, moderation, or temporal linkage between brain findings and psychological outcomes.

For studies reporting a direct neural-psychological association, we additionally charted the psychological domain, neural modality, neural measure, association model, reported coefficient or test statistic when available, *p* value or authors’ statistical-significance statement, and whether the association linked the neural measure to an eligible psychological or cognitive-emotional endpoint. Association direction was coded at the level of the eligible brain-behavior analysis as positive/favorable, null, negative/unfavorable, mixed, or not tested/no eligible direct association. “Positive/favorable” indicated that the reported neural measure was statistically associated with better psychological status, symptom reduction, or better cognitive-emotional performance, irrespective of the raw sign of the coefficient when lower scores represented better outcomes. “Null” was reserved for studies that formally tested an eligible neural-psychological association and reported no statistically significant association. Studies that only reported neural and psychological outcomes in parallel were coded separately as not tested/no eligible direct association.

### 2.7 Outcomes of interest

The primary outcome of this scoping review was the mapped landscape of studies that jointly examined Tai Chi, brain activity, and psychological outcomes. Secondary outcomes were the distribution of evidence by population, setting, Tai Chi characteristics, neural modality, psychological domain, and study design. Additional outcomes of interest included whether studies reported concurrent change in neural and psychological variables, whether formal brain-behavior associations were tested, and how studies interpreted the putative relationship between neural and psychological findings.

### 2.8 Critical appraisal and risk of bias

Risk of bias was assessed according to study design. Randomized studies were appraised using the Cochrane risk-of-bias tool for randomized trials. Non-randomized intervention studies were appraised using the Risk Of Bias In Non-randomized Studies - of Interventions tool. Analytical cross-sectional studies were appraised using the Joanna Briggs Institute critical appraisal checklist. Two authors screened and assessed independently each of the studies included. Appraisal findings were summarized descriptively at the domain level and were used to contextualize the strength, limitations, and interpretability of the included evidence within the narrative synthesis. Critical appraisal was conducted after study inclusion had been determined and was not used as an eligibility filter. All studies meeting the eligibility criteria were retained in the review, evidence tables, and evidence maps regardless of their risk-of-bias judgment.

For the narrative synthesis, risk-of-bias judgments were not used as exclusion criteria, consistent with the mapping purpose of a scoping review, but they were used to qualify the inferential weight assigned to each finding. Randomized studies judged as having some concerns were treated as relatively more informative for intervention-related inference than randomized studies judged at high risk of bias, non-randomized intervention studies judged serious overall, or cross-sectional studies judged moderate because of residual confounding and self-selection. Studies judged serious or high risk were retained in the evidence map but interpreted as exploratory or supportive rather than as primary evidence for causal or mechanistic conclusions.

### 2.9 Effect measures and synthesis methods

When individual studies reported between-group differences, within-group changes, correlations, mediation models, moderation analyses, or other inferential statistics linking neural and psychological outcomes, these were extracted and reported narratively. Because the included studies differed substantially in design, population, Tai Chi exposure, neural modality, psychological endpoint, and statistical reporting, no meta-analysis of brain-behavior association strength was attempted. Instead, we summarized association direction and consistency using a structured descriptive tables and a secondary heatmap stratified by neural modality and psychological domain, consistent with the scoping review aim of mapping evidence characteristics and gaps rather than estimating a pooled effect.

Studies were first organized by design and by neural modality, for example EEG or event-related potentials, functional MRI or resting-state MRI, fNIRS, PET, MRS, or other functional methods. Within each modality, studies were then summarized according to population type, Tai Chi exposure characteristics, psychological outcome domain, and whether the study addressed acute or longitudinal effects.

The synthesis emphasized five questions: how the literature was distributed across populations and settings; which neural and psychological outcomes had been studied most often; whether studies reported improvement, null findings, or mixed findings across the neural and psychological domains; whether investigators explicitly tested relations between neural indices and psychological outcomes; and whether eligible brain-behavior associations were reported as statistically significant favorable, null, negative/unfavorable, mixed, or not formally tested.

## 3 Results

### 3.1 Study selection

**Figure 1:**
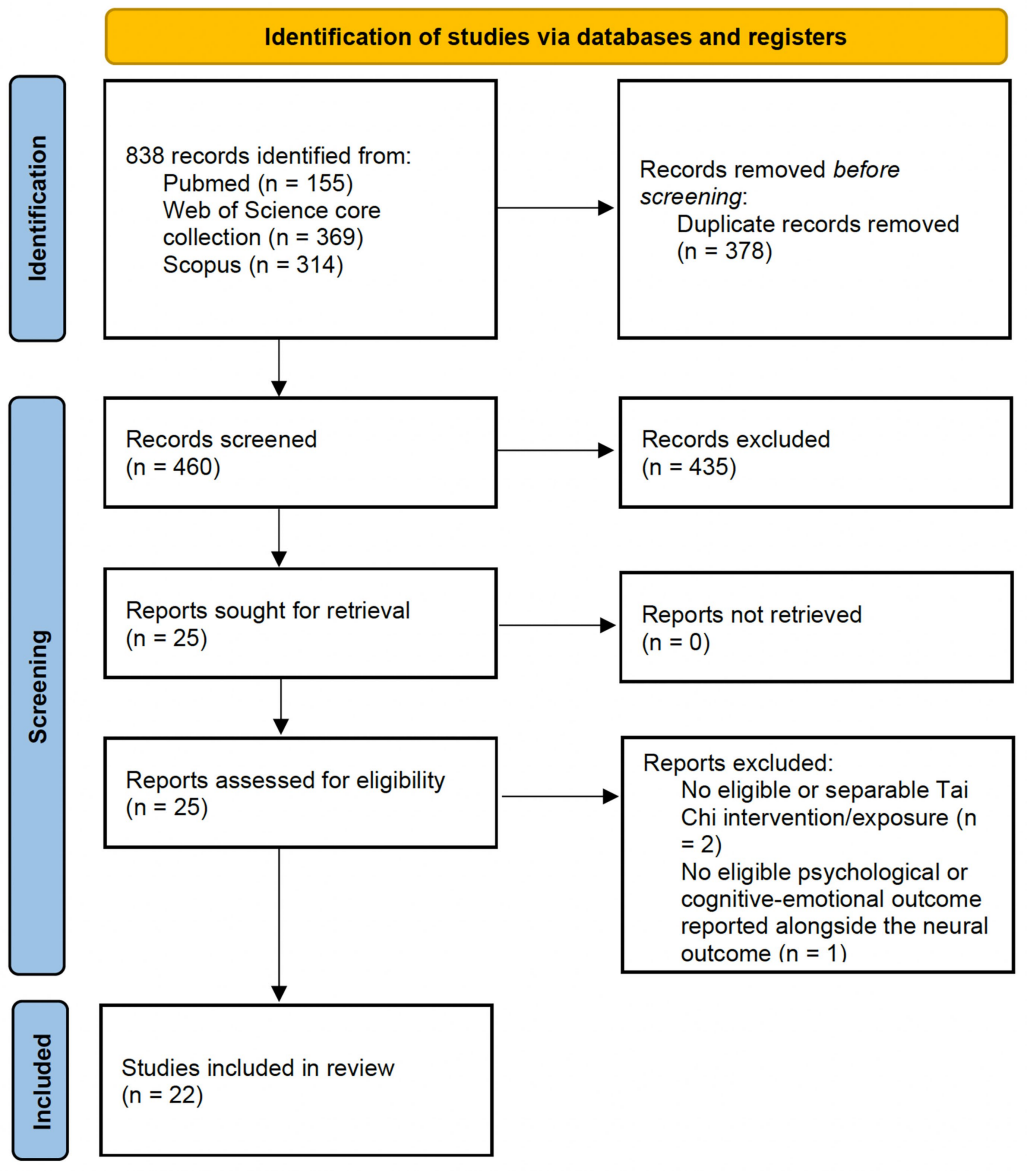
PRISMA flow diagram.

The database searches yielded 838 records in total, comprising 155 from PubMed, 369 from Web of Science Core Collection, and 314 from Scopus. After removal of 378 duplicate records, 460 unique records remained for title-and-abstract screening. Of these, 435 were excluded at the screening stage, and 25 were classified as likely eligible for full-text assessment. Twenty-two reports met the eligibility criteria and were included. Three reports were excluded at full-text assessment for prespecified eligibility reasons: two did not contain an eligible or separable Tai Chi intervention/exposure, and one did not report an eligible psychological or cognitive-emotional outcome alongside the neural outcome. Figure 1 summarizes the selection process over the phases.

### 3.2 Characteristics of included studies

Table 1 summarizes the characteristics of the 22 included studies. Older adults and university students were the most frequently studied populations, although the evidence base also included clinical cohorts with geriatric major depressive disorder, major depressive disorder, chronic fatigue syndrome, Parkinson disease, functional constipation, and type 2 diabetes mellitus with and without major depressive disorder.

**Table 1:** Characteristics of included studies examining Tai Chi, brain activity, and psychological outcomes.

Study	Population	Design	Tai Chi exposure and comparator	Exposure duration	Neural and psychological outcomes
Cheung et al. (2018)	Healthy late middle-aged Chinese adults; Tai Chi practitioners versus nonpractitioners	Prospective non-randomized controlled intervention trial	Acute self-paced Wu-style or Yang-style Tai Chi for 10 min versus quiet standing control	Acute single-session exposure	Electroencephalography-derived attention and meditation; perceived stress
He et al. (2024)	Older community-dwelling adults with sleep disturbances	Pilot randomized controlled trial	Simplified Yang-style Tai Chi alone or Tai Chi plus repetitive transcranial magnetic stimulation versus treat-as-usual	Repeated multi-session exposure over 4 weeks with 3-month follow-up	Resting-state electroencephalography cortical arousal metrics; depression, anxiety, stress, and mental-function quality of life
Ji and Li (2025)	Healthy Tai Chi-trained college students specializing in martial arts	Randomized controlled trial	Twenty-minute 24-form Yang-style Tai Chi Chuan versus twenty-minute ergo-cycle exercise or twenty-minute sitting rest after Stroop-induced mental fatigue	Acute single-session exposure	Subjective mental fatigue, Go/NoGo reaction time, and event-related potential indices of inhibitory control
Kilpatrick et al. (2022)	Older adults with geriatric major depressive disorder receiving stable antidepressant treatment	Randomized controlled neuroimaging substudy	Tai Chi Chih plus antidepressant treatment versus health education and wellness plus antidepressant treatment	Repeated multi-session exposure over 12 weeks with post-intervention follow-up	Resting-state functional connectivity, depressive symptoms, and psychological resilience
Li Y. et al. (2022)	Adults with chronic fatigue syndrome and healthy comparison participants, all receiving the same Tai Chi Chuan program	Controlled pre-post intervention study without randomization	Twenty-four-style simplified Tai Chi Chuan delivered twice weekly for 4 weeks plus daily home practice; no non-Tai-Chi comparator, but healthy controls underwent the same regimen	Repeated multi-session exposure over 4 weeks	Resting-state functional magnetic resonance imaging effective connectivity and selected 36-item Short-Form Health Survey psychological subscales
Li X. et al. (2022)	Healthy university students with routine Tai Chi practice for more than 6 months versus matched controls without Tai Chi practice	Cross-sectional controlled exploratory study	Habitual Tai Chi practice exposure versus no Tai Chi practice	Routine practice exposure for more than 6 months	Positive psychological capital, auditory oddball response time, and resting electroencephalography functional network topology
Li et al. (2024)	Adults with early-stage Parkinson disease receiving stable medication	Randomized controlled trial	Standardized Yi Tai Chi versus brisk walking versus no-exercise control	Repeated multi-session exposure over 12 months	Cognition, daytime sleepiness, non-motor symptom burden, resting-state functional magnetic resonance imaging network dynamics, and mechanistic blood biomarkers
Li et al. (2025)	Community-recruited older adults without major neurodegenerative disease	Randomized controlled trial	Groove-music-integrated Tai Chi versus conventional-music Tai Chi versus low-intensity activity control	Repeated multi-session exposure over 12 weeks	Emotional regulation, executive function, Stroop inhibitory control, and prefrontal functional near-infrared spectroscopy connectivity
Liu et al. (2018)	Healthy older adults with long-term Tai Chi experience and active non-meditative controls	Cross-sectional study	Routine long-term Tai Chi practice versus other physical exercise without meditation components	Routine practice exposure	Emotion-regulation sensitivity, mindfulness-related non-judgment, and resting-state executive-control-network connectivity
Liu et al. (2020)	Healthy older adults with long-term Tai Chi experience and active non-meditative controls	Cross-sectional study	Routine long-term Tai Chi practice versus other physical exercise without meditation components	Routine practice exposure	Regret-related emotional sensitivity, mindfulness-related non-judgment, risk taking, and task-based fronto-striatal connectivity
Port et al. (2018)	Healthy older Tai Chi and Water Aerobics practitioners	Case–control study	Routine long-term Tai Chi practice versus routine Water Aerobics practice	Routine practice exposure	Anxiety, neuropsychological test battery, and task-based functional magnetic resonance imaging during attention and working-memory tasks
Teng et al. (2025)	Adults with functional constipation, with healthy subjects recruited as a baseline imaging reference cohort	Randomized controlled trial	Structured Tai Chi versus aerobic exercise, with additional healthy reference subjects for baseline brain comparison	Repeated multi-session exposure over 8 weeks	Constipation symptom severity, anxiety, depression, heart-rate variability, and resting-state anterior-insula functional connectivity within the central autonomic network
Wang et al. (2007)	Healthy middle-elderly adults with prior 24-style Taijiquan experience, subdivided by self-reported use of “Taiji sense” imagery and compared with an ergometry comparator group	Nonrandomized acute three-group psycho-physiological study	Taiji-sensed 24-style Taijiquan versus non-Taiji-sensed 24-style Taijiquan versus heart-rate-matched ergometry exercise	Acute single-session exposure	Profile of Mood States tension-anxiety and vigor outcomes; resting electroencephalography alpha, beta, and frontal asymmetry outcomes
Wang et al. (2024)	Healthy college students during the COVID-19 pandemic	Randomized controlled trial	Structured Tai Chi versus routine daily activities without additional structured exercise	Repeated multi-session exposure over 12 weeks	State and trait anxiety, total State–Trait Anxiety Inventory score, and resting-state electroencephalography theta oscillatory power
Wang et al. (2025)	Healthy university students without psychiatric disorders or medication use	Article-described within-subjects crossover acute exercise study	Acute Eight Methods and Five Steps Tai Chi versus matched-intensity power cycling versus quiet-rest control	Acute single-session exposure	Positive emotional memory accuracy and task-based left dorsolateral prefrontal cortex oxygenated-hemoglobin concentration
Wu et al. (2022)	Adults with chronic fatigue syndrome plus matched healthy controls, all Tai Chi-naïve at baseline	Longitudinal controlled pre-post study without randomization	One month of simplified 24-style Tai Chi in both chronic fatigue syndrome and healthy-control cohorts	Repeated multi-session exposure over four weeks	Fatigue, sleep quality, overall SF-36 health state, and resting-state network connectivity with machine-learning-derived functional connections
Xu et al. (2020)	Chinese American adults with major depressive disorder	Pilot single-group pre-post study	Ten weeks of Yang-style Tai Chi without a control group	Repeated multi-session exposure over ten weeks	Beck Depression Inventory, SF-36 vitality subscale, and resting-state insular functional connectivity
Yuan et al. (2025)	Healthy female college students (20 Tai Chi, 20 control)	Randomized parallel-group trial	Eight-week Tai Chi training, 3 sessions per week, 50 min per session, versus no-intervention control with matched observation/contact schedule	Repeated multi-session exposure over 8 weeks	Event-related potential N2 and P3 during modified oddball emotional-processing task; reaction time and emotional-regulation / emotional-processing behavioral performance
Zhang et al. (2022)	Older adults with long-term Tai Chi practice, older walking group, older non-exercise group, and contextual younger controls	Analytical cross-sectional comparative study	Long-term Tai Chi exercise habit (at least 5 years, more than 1 h per day and more than 5 days per week) versus older walking and older non-exercise comparison groups	Single assessment of long-term prior exposure	Emotional-face recognition and memory accuracy / reaction time; event-related potential measures during learning and test phases for negative and neutral faces
Zhang et al. (2023)	College students with anxiety and depression (9 Tai Chi, 9 control)	Randomized parallel-group pilot trial	Eight-week Bafa Wubu of Tai Chi, 5 sessions per week, 60 min per session, versus usual daily life without exercise intervention	Repeated multi-session exposure over 8 weeks	Resting-state functional magnetic resonance imaging fractional amplitude of low-frequency fluctuations; self-rating anxiety scale; self-rating depression scale
Zhang et al. (2024)	Older adults with type 2 diabetes mellitus and major depressive disorder, adults with type 2 diabetes mellitus without major depressive disorder, and healthy controls; all Tai Chi-naive	Analytical cross-sectional task-based comparative study	Standardized Tai Chi Chuan motor task after 1-week instruction, used as the task during functional near-infrared spectroscopy acquisition	Single cross-sectional assessment after brief familiarization	Functional near-infrared spectroscopy activation, functional connectivity, and lateralization in prefrontal and motor areas; Hamilton Depression Rating Scale-24 depressive symptoms
Zhao et al. (2025)	Healthy older adults aged 60 to 70 years	Randomized repeated-measures / crossover-like study with separable Tai Chi-containing conditions	Taijiquan-alone condition and Five-Element Music plus Taijiquan condition, each compared within participants against baseline and non-Tai-Chi conditions	Baseline plus five conditions with 1-week washout between tests	Emotion-regulation choice task for high-intensity unpleasant pictures; two-back working-memory performance; functional near-infrared spectroscopy functional connectivity

Eleven studies were randomized or condition-randomized intervention studies, five were non-randomized intervention studies, and six were cross-sectional or case–control comparisons. Eleven studies evaluated repeated multi-session interventions, five examined routine long-term Tai Chi practice as the exposure of interest, and six used acute, task-based, or repeated-measures session paradigms. Most studies evaluated Tai Chi as a stand-alone intervention or exposure, but several studies included separable Tai Chi-containing multicomponent conditions, particularly Tai Chi combined with repetitive transcranial magnetic stimulation, groove music, or Five-Element Music.

Eight included studies used electroencephalography or event-related potentials, ten used functional magnetic resonance imaging when resting-state and task-based paradigms were considered together, and four used functional near-infrared spectroscopy. Psychological outcomes were broad and often multidomain, spanning stress, depression, anxiety, fatigue, mood or affect, mental health-related quality of life and well-being, and other cognitive-emotional or neuropsychological endpoints such as emotion regulation, emotional memory, working memory, inhibitory control, and emotional-face recognition. Table 1 presents the characteristics of all 22 included studies.

### 3.3 Critical appraisal of included evidence

Among randomized parallel-group and repeated-measures studies, the predominant judgment was some concerns (Table 2). The most frequent factors were incomplete reporting of allocation concealment or sequence concealment, unavoidable lack of participant blinding for movement interventions, reliance on self-reported psychological outcomes, attrition or artifact-related loss of neural data, and limited information on prespecified analysis hierarchies for multivariate neural outcomes.

**Table 2:** Appraisal of randomized studies using the Cochrane risk of bias 2 tool.

Study	Randomization process	Deviations from intended interventions	Missing outcome data	Outcome measurement	Reported-result selection	Overall judgment
He et al. (2024)	Some concerns	Some concerns	Some concerns	Some concerns	Some concerns	Some concerns
Ji and Li (2025)	Some concerns	Some concerns	Some concerns	Some concerns	Some concerns	Some concerns
Kilpatrick et al. (2022)	Some concerns	Some concerns	Some concerns	Some concerns	Some concerns	Some concerns
Li et al. (2024)	Some concerns	Some concerns	High	Some concerns	Some concerns	High
Li et al. (2025)	Some concerns	Some concerns	Some concerns	Some concerns	Some concerns	Some concerns
Teng et al. (2025)	Some concerns	Some concerns	Some concerns	Some concerns	Some concerns	Some concerns
Wang et al. (2024)	Some concerns	Low	Low	Some concerns	Some concerns	Some concerns
Yuan et al. (2025)	Some concerns	Low	Some concerns	Low	Some concerns	Some concerns
Zhang et al. (2023)	Some concerns	Low	Low	Some concerns	Some concerns	Some concerns
Wang et al. (2025)	Some concerns	Some concerns	Some concerns	Some concerns	Low	Some concerns
Zhao et al. (2025)	Some concerns	Some concerns	Some concerns	Low	Low	Some concerns

All the non-randomized intervention studies appraised with the Risk Of Bias In Non-randomized Studies - of Interventions tool were judged serious overall (Table 3). The principal limitation was confounding, usually because participants were allocated by prior practice history, because all groups received Tai Chi without a concurrent non-Tai-Chi control, or because single-group pre-post designs could not separate intervention effects from time, expectancy, or regression to the mean. The cross-sectional studies were appraised as moderate (Table 4), mainly because exposure was self-selected and confounding was insufficiently addressed despite otherwise acceptable outcome measurement and reporting. These appraisal judgments were used only to contextualize interpretation. No study was excluded from the review, evidence tables, or evidence maps because of its risk-of-bias rating. Accordingly, studies judged high risk, serious risk, or moderate risk were retained in the descriptive synthesis, but their findings were interpreted with appropriately reduced inferential weight.

**Table 3:** Appraisal of non-randomized intervention studies using the risk of bias in non-randomized studies - of interventions tool.

Study	Confounding	Selection of participants	Classification of intervention	Deviations from intended interventions	Missing data	Outcome measurement	Reported-result selection	Overall judgment
Cheung et al. (2018)	Serious	Moderate	Low	Low	Low	Serious	Moderate	Serious
Li Y. et al. (2022)	Serious	Moderate	Low	Moderate	Low	Moderate	Moderate	Serious
Wang et al. (2007)	Serious	Moderate	Moderate	Low	Low	Moderate	Moderate	Serious
Wu et al. (2022)	Serious	Moderate	Low	Moderate	Low	Moderate	Moderate	Serious
Xu et al. (2020)	Serious	Moderate	Low	Moderate	Low	Moderate	Moderate	Serious

**Table 4:** Appraisal of cross-sectional studies using the Joanna Briggs Institute critical appraisal checklist for analytical cross-sectional studies.

Study	Inclusion criteria defined	Subjects and setting described	Exposure measured validly	Objective condition criteria used	Confounding identified	Confounding addressed	Outcome measurement valid	Statistical analysis appropriate	Overall judgment
Li X. et al. (2022)	Yes	Yes	Yes	Unclear	No	No	Yes	Yes	Moderate
Liu et al. (2018)	Yes	Yes	Yes	Unclear	Yes	Yes	Yes	Yes	Moderate
Liu et al. (2020)	Yes	Yes	Yes	Unclear	Yes	Yes	Yes	Yes	Moderate
Port et al. (2018)	Yes	Yes	Yes	Unclear	Yes	Yes	Yes	Yes	Moderate
Zhang et al. (2022)	Yes	Yes	Yes	Unclear	No	No	Yes	Yes	Moderate
Zhang et al. (2024)	Yes	Yes	Yes	Yes	Unclear	No	Yes	Yes	Moderate

### 3.4 Results of individual studies

Table 5 presents a summary of the principal neural findings, psychological or cognitive-emotional findings, and any reported brain-behavior associations for all included studies. To address the direction and consistency of these associations more explicitly, Table 6 summarizes study eligible brain-behavior association findings by neural modality, psychological domain, association direction, and statistical support.

**Table 5:** Main results of individual studies examining Tai Chi, brain activity, and psychological outcomes.

Study	Neural and psychological outcomes	Main results and brain-behavior association	Risk-of-bias-informed interpretation
Cheung et al. (2018)	Electroencephalography-derived attention and meditation; perceived stress	Transient increase in electroencephalography-derived attention during Tai Chi and reduced perceived stress after Tai Chi, however no significant meditation effect was found.	Exploratory evidence only. The findings support a possible short-term psychophysiological response during Tai Chi, but they should not be interpreted as evidence of a causal training effect or durable brain-behavior mechanism.
He et al. (2024)	Resting-state electroencephalography cortical arousal metrics; depression, anxiety, stress, and mental-function quality of life	Both Tai Chi-containing arms improved depression, stress, and mental-function quality of life versus treat-as-usual; anxiety interaction not significant; and electroencephalography mediation did not explain insomnia change.	Some concerns. Because the eligible psychological improvements were not explained by the electroencephalography mediation model, and because one arm combined Tai Chi with repetitive transcranial magnetic stimulation, this study supports psychological benefit in Tai Chi-containing conditions but provides limited direct evidence for brain-behavior coupling.
Ji and Li (2025)	Subjective mental fatigue, Go/NoGo reaction time, and event-related potential indices of inhibitory control	Tai Chi Chuan reduced subjective mental fatigue versus sitting rest and showed within-group recovery of several event-related potential indices after mental-fatigue induction; reaction-time interaction was not statistically significant.	The overall judgment was some concerns. The findings support an acute fatigue-recovery signal, but the absence of a direct eligible brain-behavior association limits mechanistic inference.
Kilpatrick et al. (2022)	Resting-state functional connectivity, depressive symptoms, and psychological resilience	Tai Chi Chih produced more widespread connectivity increases than the active comparator, especially involving the default mode network. Geriatric Depression Scale improvement was greater with Tai Chi Chih, whereas Hamilton Depression Rating Scale and resilience between-group changes were not significant. Connectivity increases were positively associated with depressive-symptom and resilience improvement.	Overall judgment was some concerns and the neuroimaging sample was limited. The association findings should be interpreted as cautious support for brain-behavior coupling rather than definitive evidence of mediation.
Li Y. et al. (2022)	Resting-state functional magnetic resonance imaging effective connectivity and selected 36-item Short-Form Health Survey psychological subscales	The chronic fatigue syndrome cohort showed significant improvement in vitality and mental-health subscales after Tai Chi Chuan. Resting-state effective connectivity between the sensorimotor network and default mode network was abnormally weakened at baseline in chronic fatigue syndrome and was enhanced after training.	Exploratory evidence only. All groups received Tai Chi without a clearly isolating non-Tai-Chi comparator. The findings suggest temporal convergence between symptom improvement and connectivity change but cannot establish Tai Chi-specific effects or direct neural mediation of psychological outcomes.
Li X. et al. (2022)	Positive psychological capital, auditory oddball response time, and resting electroencephalography functional network topology	Routine Tai Chi practitioners showed higher positive psychological capital, faster response time, stronger alpha-band network efficiency and clustering, shorter path length, and greater frontal-temporal node degree than controls. The study also reported neural correlations with questionnaire score and with response time.	The cross-sectional study was judged moderate, with residual confounding because Tai Chi exposure was self-selected. The neural-psychological correlations are useful for identifying candidate signatures of long-term practice exposure, but they cannot determine whether Tai Chi caused the observed psychological or network differences.
Li et al. (2024)	Cognition, daytime sleepiness, non-motor symptom burden, resting-state functional magnetic resonance imaging network dynamics, and mechanistic blood biomarkers	Tai Chi improved Parkinson’s Disease Cognitive Rating Scale total and frontal cortical scores versus no-exercise control at 12 months and showed earlier advantages for overall non-motor symptom burden and daytime sleepiness. Enhanced somatomotor-network function and favorable cytokine, metabolomic, and Huntingtin interaction protein 2 changes were associated with cognitive improvement; depression, anxiety, fatigue, and quality-of-life scales were not significantly different.	Judged high risk overall. Its cognitive and neural findings may inform hypothesis development, but they should not be given the same inferential weight as randomized studies judged as having some concerns, and they do not provide strong support for eligible psychological brain-behavior effects because depression, anxiety, fatigue, and quality-of-life outcomes were not clearly improved.
Li et al. (2025)	Emotional regulation, executive function, Stroop inhibitory control, and prefrontal functional near-infrared spectroscopy connectivity	Groove-music-integrated Tai Chi improved low-intensity emotional regulation, working memory, planning, and Stroop word interference more than control, with some advantages over conventional-music Tai Chi. Functional near-infrared spectroscopy showed stronger medial-prefrontal to left-prefrontal connectivity after groove-music Tai Chi, while the study reported additional ancillary electromyography coherence findings as a possible mechanism.	Some concerns. The findings support prefrontal involvement in a Tai Chi-containing intervention, but they do not fully isolate the Tai Chi-specific contribution or establish a direct eligible brain-behavior association.
Liu et al. (2018)	Emotion-regulation sensitivity, mindfulness-related non-judgment, and resting-state executive-control-network connectivity	Tai Chi practitioners showed greater non-judgment of inner experience, less emotional sensitivity to outcomes, and weaker dorsolateral-prefrontal to middle-frontal resting-state connectivity than controls. The dorsolateral-prefrontal to middle-frontal connectivity fully mediated the relationship between non-judgment and emotion-regulation ability.	Judged as moderate. The finding should be framed as a candidate brain-behavior pathway requiring prospective confirmation.
Liu et al. (2020)	Regret-related emotional sensitivity, mindfulness-related non-judgment, risk taking, and task-based fronto-striatal connectivity	Tai Chi practitioners showed less regret-related emotional sensitivity, lower risk taking, and higher non-judgment of inner experience than controls. Task-based functional magnetic resonance imaging showed stronger fronto-striatal connectivity in poor-outcome trials, and non-judgment fully mediated the relationship between this connectivity pattern and reduced emotional sensitivity to outcome.	Judged as moderate. The brain-behavior mediation model is useful for identifying a plausible fronto-striatal pathway, but causal interpretation should be avoided because long-term Tai Chi exposure was self-selected and temporality was not established.
Port et al. (2018)	Anxiety, neuropsychological test battery, and task-based functional magnetic resonance imaging during attention and working-memory tasks	Tai Chi and Water Aerobics practitioners showed similar neuropsychological performance, but the Tai Chi group had lower anxiety. During Stroop and N-back tasks, the Tai Chi group showed lower task-related activation in occipital and frontal regions, suggesting a greater neural efficiency.	Judged as moderate. Lower anxiety and lower task-related activation were observed in Tai Chi practitioners, but no direct eligible brain-anxiety association was tested, and the design cannot distinguish Tai Chi-related adaptation from pre-existing group differences or self-selection.
Teng et al. (2025)	Constipation symptom severity, anxiety, depression, heart-rate variability, and resting-state anterior-insula functional connectivity within the central autonomic network	Both Tai Chi and aerobic exercise improved constipation symptoms, mood, and autonomic balance. Tai Chi showed a modest advantage for constipation symptom severity and uniquely increased left-anterior-insula connectivity with anterior cingulate and posterior-insula regions. These neural changes were linked to symptom reduction and parasympathetic indices, whereas anxiety and depression did not differ between exercise groups.	Judge as some concerns. The neural associations primarily support autonomic and constipation-related mechanisms rather than eligible psychological brain-behavior coupling, since anxiety and depression did not show clear between-group advantages.
Wang et al. (2007)	Profile of Mood States tension-anxiety and vigor outcomes; resting electroencephalography alpha, beta, and frontal asymmetry outcomes	All exercise conditions lowered tension-anxiety, but only the Taiji-sensed Tai Chi subgroup showed increased vigor. Relative alpha power increased more and beta power decreased more after Taiji-sensed Tai Chi than after ergometry, and alpha change correlated strongly with tension-anxiety change.	Exploratory psychophysiological evidence only. Judged as serious overall, with important confounding risk related to group classification and prior experience. The alpha–tension-anxiety correlation is relevant for brain-behavior mapping but should not be treated as causal evidence that Tai Chi reduced anxiety through alpha-power modulation.
Wang et al. (2024)	State and trait anxiety, total State–Trait Anxiety Inventory score, and resting-state electroencephalography theta oscillatory power	Tai Chi reduced anxiety scores whereas the control group worsened over time. Theta oscillatory power increased across several frontal, central, parietal, and temporal electrodes in the Tai Chi group, and greater theta increases were associated with larger anxiety reductions.	The overall judgment was some concerns. The finding provides cautious support for EEG-indexed brain-behavior coupling in anxiety, while remaining vulnerable to self-report outcome limitations and multiplicity in electrode-level neural analyses.
Wang et al. (2025)	Positive emotional memory accuracy and task-based left dorsolateral prefrontal cortex oxygenated-hemoglobin concentration	Tai Chi produced the clearest improvement in positive emotional memory and the strongest increase in left dorsolateral prefrontal activation, with a positive brain-behavior correlation.	Judged as having some concerns. The direct association between left dorsolateral prefrontal activation and positive emotional memory supports a plausible acute prefrontal mechanism, but it should not be generalized to long-term psychological adaptation.
Wu et al. (2022)	Fatigue, sleep quality, overall SF-36 health state, and resting-state network connectivity with machine-learning-derived functional connections	Fatigue improved significantly in the chronic fatigue syndrome cohort and abnormal default mode / left frontoparietal network patterns shifted toward healthy-control values after Tai Chi.	Judged as serious overall. The findings suggest temporal normalization of network features alongside fatigue improvement, but the design cannot isolate Tai Chi-specific effects from time, expectancy, repeated testing, or regression to the mean.
Xu et al. (2020)	Beck Depression Inventory, SF-36 vitality subscale, and resting-state insular functional connectivity	Depressive symptoms decreased and vitality increased after Tai Chi. Distinct right-insular connectivity changes were linked to mood versus vitality improvement.	Judged as serious overall because it lacked a concurrent control group. The insular-connectivity associations are valuable for hypothesis generation but cannot establish that Tai Chi caused the symptom or connectivity changes.
Yuan et al. (2025)	Event-related potential N2 and P3 during modified oddball emotional-processing task; reaction time and emotional-regulation / emotional-processing behavioral performance	After 8 weeks, the Tai Chi group showed shorter reaction times than the control group. Neural findings included lower N2 amplitudes to negative stimuli, shorter overall N2 latencies, and exploratory increases in P3 amplitude across valence conditions.	Overall judgment was some concerns. The findings suggest improved emotional-processing efficiency after Tai Chi. Results should be interpreted as parallel outcomes rather than evidence of mechanistic coupling.
Zhang et al. (2022)	Emotional-face recognition and memory accuracy / reaction time; event-related potential measures during learning and test phases for negative and neutral faces	The Tai Chi and walking groups showed better learning-phase emotional-face recognition accuracy than older non-exercise controls, but Tai Chi did not show a clear event-related-potential advantage over older controls in the reported later mean-amplitude analyses. In the test phase, the Tai Chi group did not outperform older non-exercise controls in emotional-face memory accuracy and performed worse than the walking group on some memory outcomes.	Judged as moderate, and long-term exercise exposure was self-selected. Because Tai Chi did not consistently outperform comparator groups and no direct eligible brain-behavior association was established, this study should temper rather than strengthen claims about Tai Chi-specific ERP advantages in emotional-face processing.
Zhang et al. (2023)	Resting-state functional magnetic resonance imaging fractional amplitude of low-frequency fluctuations; self-rating anxiety scale; self-rating depression scale	After the intervention, anxiety and depression scores decreased in the Tai Chi group. Fractional amplitude of low-frequency fluctuations increased in the right middle frontal gyrus orbital part, right inferior occipital gyrus, and right middle temporal gyrus temporal pole, and decreased in the left middle frontal gyrus and right supplementary motor area. Right orbitofrontal activity correlated positively with depression scores, whereas left middle frontal activity correlated negatively with anxiety scores.	The overall judgment was some concerns. The association pattern should be interpreted as mixed rather than uniformly favorable, particularly because the direction of symptom scales and regional activity associations requires cautious interpretation. This study supports further testing rather than firm conclusions about fMRI markers of anxiety or depression improvement.
Zhang et al. (2024)	Functional near-infrared spectroscopy activation, functional connectivity, and lateralization in prefrontal and motor areas; Hamilton Depression Rating Scale-24 depressive symptoms	Compared with the type 2 diabetes mellitus and healthy groups, the type 2 diabetes mellitus plus major depressive disorder group showed reduced activation, abnormal lateralization, and lower functional connectivity among supplementary motor, motor, and dorsolateral prefrontal regions during the standardized Tai Chi task. Dorsolateral prefrontal oxyhaemoglobin concentration was inversely associated with depressive-symptom severity.	Judged as moderate. The inverse association between dorsolateral prefrontal oxygenation and depressive-symptom severity is relevant for identifying candidate fNIRS markers, but the design involved a single task-based assessment after brief familiarization and cannot establish intervention-induced change.
Zhao et al. (2025)	Emotion-regulation choice task for high-intensity unpleasant pictures; two-back working-memory performance; functional near-infrared spectroscopy functional connectivity	The strongest reported benefits were observed in the Five-Element Music plus Taijiquan condition, which improved acceptance of high-intensity unpleasant pictures, increased two-back accuracy, and strengthened prefrontal-parietal functional connectivity. Five-Element Music alone, rather than Taijiquan alone, was associated with faster two-back reaction time. The Taijiquan-alone condition was retained as an eligible evidence unit for mapping, but it was not singled out in the reported pairwise contrasts as the strongest condition.	Judged as having some concerns. However, interpretation for Tai Chi specifically should be cautious because the strongest findings occurred in the combined Five-Element Music plus Taijiquan condition and no direct eligible brain-behavior association for the Taijiquan-alone condition was clearly established.

**Table 6:** Direction of eligible brain-behavior associations by neural modality and psychological domain.

Study	Modality	Psychological domain	Association classification	Evidence
Kilpatrick et al. (2022)	rs-fMRI	Depression, resilience	Significant favorable	Connectivity latent variables associated with HAMD, GDS, and resilience improvement (r values approximately 0.53–0.64 in Tai Chi).
Li X. et al. (2022)	EEG	Mental well-being, cognitive-emotional performance	Significant favorable	Frontal-temporal node degree correlated with positive psychological capital; network topology correlated with response time.
Li et al. (2024)	rs-fMRI	Cognitive-emotional/neuropsychological endpoint	Significant favorable	Somatomotor-network enhancement associated with cognitive improvement; depression, anxiety, fatigue, and QoL did not show clear between-group advantages.
Liu et al. (2018)	rs-fMRI	Emotion regulation / non-judgment	Significant favorable mediation	Executive-control connectivity mediated the relation between non-judgment and emotion-regulation ability.
Liu et al. (2020)	task fMRI	Regret-related emotional sensitivity	Significant favorable mediation	Fronto-striatal connectivity and non-judgment were linked to reduced regret-related emotional sensitivity.
Wang et al. (2007)	EEG	Anxiety	Significant favorable	Alpha-power change inversely correlated with tension-anxiety change (r = −0.78, *p* < 0.0001).
Wang et al. (2024)	EEG	Anxiety	Significant favorable	Theta increases were associated with larger anxiety reductions.
Wang et al. (2025)	fNIRS	Emotional memory	Significant favorable	Left dorsolateral prefrontal oxyhemoglobin associated with positive emotional-memory accuracy (r = 0.723, *p* < 0.001).
Xu et al. (2020)	rs-fMRI	Depression, vitality/fatigue	Significant favorable	Distinct right-insular connectivity changes were linked to depression and vitality improvement.
Zhang et al. (2023)	rs-fMRI	Anxiety, depression	Mixed	Left middle-frontal activity correlated negatively with anxiety scores, whereas right orbitofrontal activity correlated positively with depression scores.
Zhang et al. (2024)	fNIRS	Depression	Significant favorable	Dorsolateral prefrontal oxyhemoglobin concentration was inversely associated with depressive-symptom severity.

At the study level, a minority of included studies reported statistically significant eligible brain-behavior associations, whereas many studies measured neural and psychological outcomes in parallel without testing a direct eligible association. Statistically significant favorable associations were reported in several functional magnetic resonance imaging studies, including associations involving depressive symptoms, resilience or vitality, emotion-regulation outcomes, and cognitive-emotional performance. In electroencephalography/event-related-potential studies, favorable associations were most apparent for anxiety or cognitive-emotional endpoints. In functional near-infrared spectroscopy studies, direct favorable associations were limited to emotional-memory or depression-related prefrontal findings. No study provided consistent evidence of a reproducible negative or adverse brain-behavior association pattern across modalities. However, one study reported mixed symptom-severity correlations, and several studies reported neural change and psychological improvement without a formal eligible neural-psychological association test.

### 3.5 Evidence map and evidence gaps

**Figure 2:**
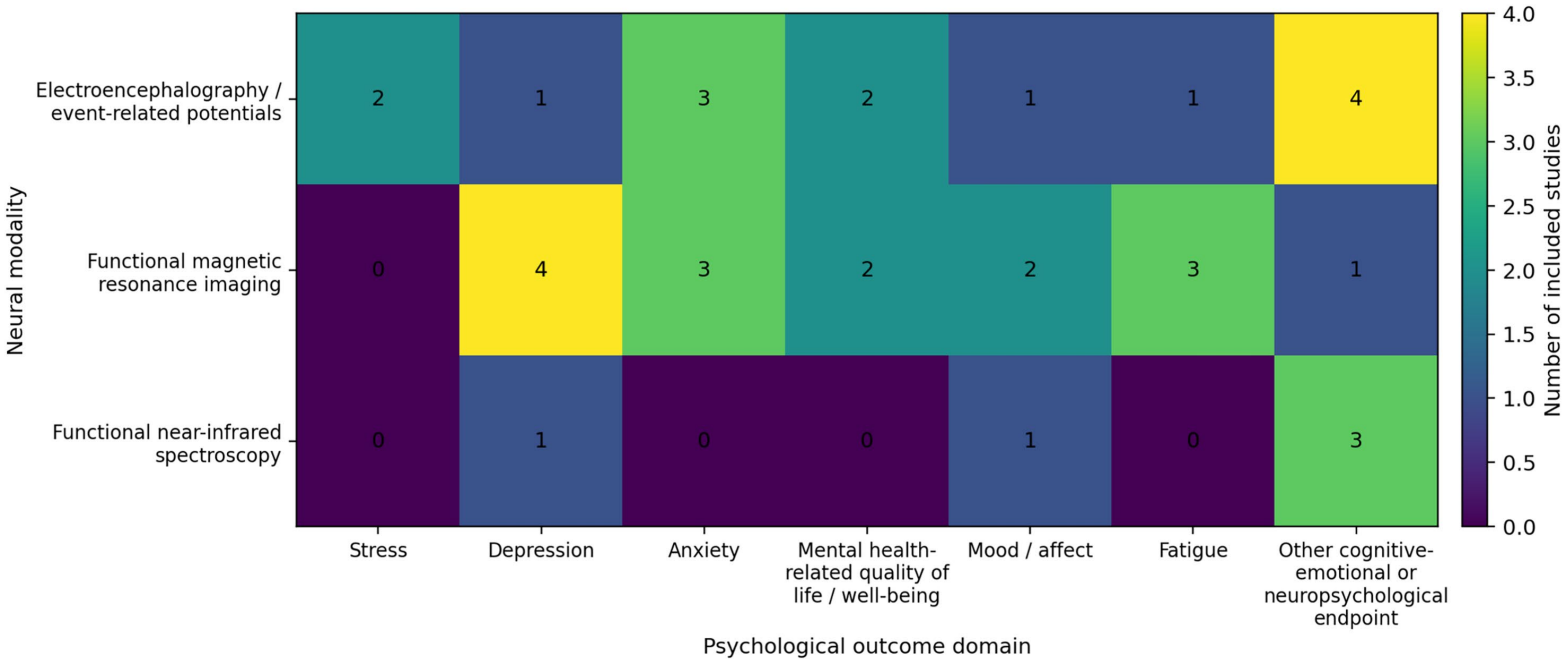
Evidence gap map of neural modality by psychological outcome domain. Each cell shows the number of included studies contributing at least one outcome within that modality-domain combination.

An evidence gap map is shown in Figure 2. The densest clusters were electroencephalography or event-related-potential studies addressing other cognitive-emotional or neuropsychological endpoints. A second major cluster comprised functional magnetic resonance imaging studies addressing depression and fatigue or vitality-related outcomes. The functional near-infrared spectroscopy cluster remained smaller, although it extended beyond emotional-memory paradigms to include depression-related task findings and repeated-measures emotion-regulation and working-memory evidence.

Stress outcomes were confined to electroencephalography studies. Mental health-related quality of life or well-being outcomes were represented mainly in electroencephalography and functional magnetic resonance imaging studies and were not meaningfully represented in functional near-infrared spectroscopy studies. Anxiety remained uncommon in functional near-infrared spectroscopy work, and no included study used positron-emission tomography or magnetic resonance spectroscopy.

**Figure 3:**
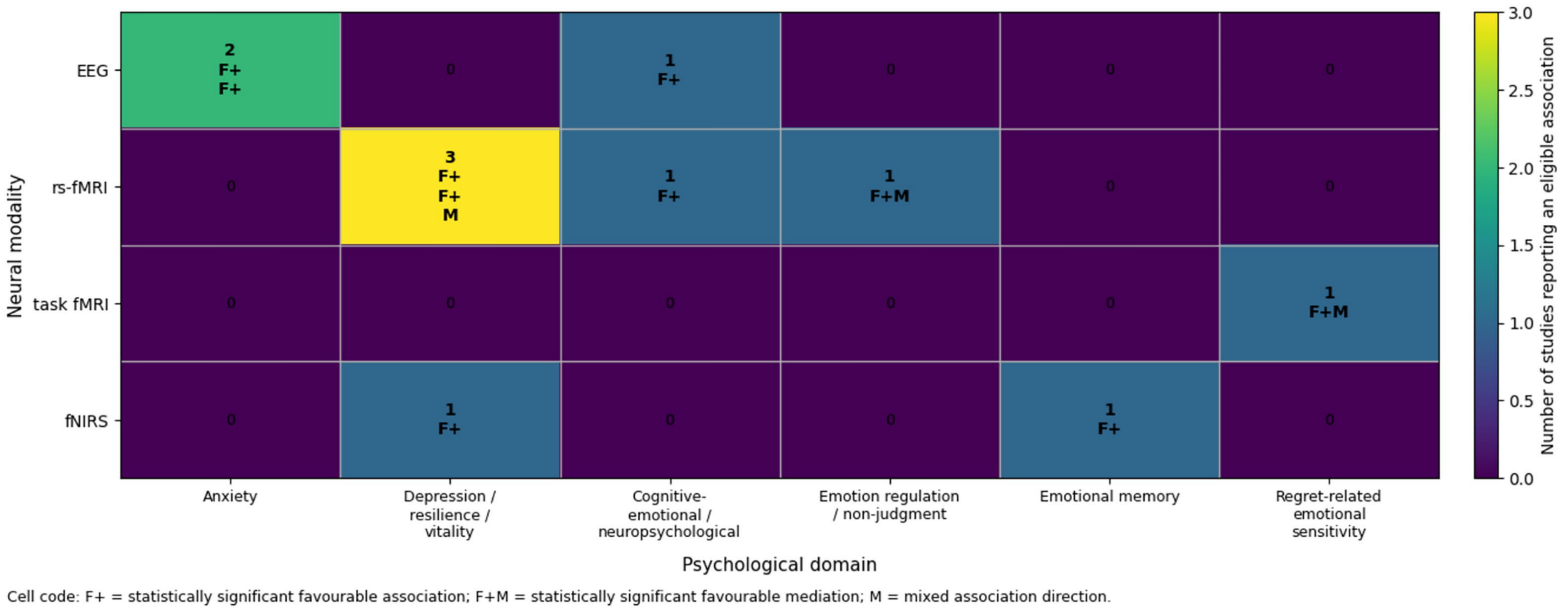
Brain–behavior association map in Tai Chi studies. Cells show the number of studies reporting an eligible direct brain–behavior association within each neural-modality and psychological-domain combination. F + indicates a statistically significant favorable association; F + M indicates a statistically significant favorable mediation finding; M indicates mixed association direction. Counts are based only on studies in which an eligible neural–psychological association was reported, and therefore do not include studies that measured neural and psychological outcomes in parallel without testing a direct association.

Figure 3 indicates whether studies within each modality-domain cell reported statistically significant favorable, null, negative/unfavorable, mixed, or not-tested/no eligible direct brain-behavior associations. The association-direction map showed that the presence of studies within a modality-domain cell did not necessarily imply evidence for a direct neural-psychological association.

## 4 Discussion

This systematic scoping review was undertaken to identify and map human studies that jointly examined Tai Chi, at least one measure of brain activity or brain function, and at least one psychological outcome. The mapped literature indicates that this field has expanded beyond the narrower neuroimaging-focused evidence base summarized in earlier reviews, which was dominated by studies in healthy older adults and by electroencephalography and functional magnetic resonance imaging methods (Pan et al., 2018). In the present review, the evidence remained methodologically and clinically heterogeneous, but it was no longer confined to a single population or a single methodological tradition. Instead, the literature now spans healthy university students, community-dwelling older adults, and several clinical groups. These clinical groups include people with geriatric depression, major depressive disorder, chronic fatigue syndrome, Parkinson disease, and functional constipation (Cheung et al., 2018; Kilpatrick et al., 2022; Li X. et al., 2022; Li et al., 2024; Teng et al., 2025; Wang et al., 2024; Xu et al., 2020). This aspect is important because it shows that the intersection between Tai Chi, neural measures, and psychological outcomes has become sufficiently developed to warrant structured evidence mapping, even though it is not yet sufficiently homogeneous for a single pooled effectiveness estimate.

A central contribution of this review is that it brings together two lines of literature that have often been synthesized separately. Previous reviews have addressed Tai Chi in relation to anxiety, depression, stress, and broader psychological well-being across heterogeneous populations, but they have generally not integrated concurrent neural measures into those syntheses (Kraft et al., 2024; Kuang et al., 2024; Sani et al., 2023; Wang et al., 2010; Wang et al., 2014; Zhu et al., 2024). Conversely, neuroimaging-oriented summaries have tended to focus on neural outcomes without systematically mapping which psychological domains were measured in parallel and whether brain-behavior links were explicitly tested (Pan et al., 2018). The present review therefore contributes a more integrated picture of the field. Across the included studies, the most recurrent overall pattern was not a uniform intervention effect, but rather a recurring co-occurrence of favorable psychological or cognitive-emotional findings with contemporaneous changes in neural connectivity, oscillatory activity, event-related potentials, or prefrontal hemodynamics. At the same time, that convergence was uneven, frequently exploratory, and highly dependent on study design, population, comparator, and modality.

### 4.1 Modality and population patterns

Electroencephalography and event-related-potential studies were concentrated in acute paradigms, educational settings, or routine-practice comparisons. Functional magnetic resonance imaging studies were more common in older-adult and clinical populations. Functional near-infrared spectroscopy represented a smaller but clearly emerging prefrontal cluster. Electroencephalography has been used to examine relatively immediate state-related shifts in stress, anxiety, mental fatigue, attention, inhibitory control, or mood-related processing during or shortly after Tai Chi exposure (Cheung et al., 2018; He et al., 2024; Ji and Li, 2025; Wang et al., 2007; Wang et al., 2024). Functional magnetic resonance imaging, by contrast, has been used more often to interrogate network-level changes in default mode, insular, executive-control, fronto-striatal, and somatomotor systems in relation to depression, fatigue, emotional regulation, and non-motor symptom profiles over repeated practice or long-term exposure (Kilpatrick et al., 2022; Li X. et al., 2022; Li et al., 2024; Liu et al., 2018; Liu et al., 2020; Port et al., 2018; Teng et al., 2025; Wu et al., 2022; Xu et al., 2020). Functional near-infrared spectroscopy studies have focused mainly on prefrontal activation or connectivity during emotional-memory, working-memory, or emotion-regulation tasks, thereby opening a potentially useful line of inquiry for movement-compatible and more ecologically oriented paradigms (Li et al., 2025; Wang et al., 2025; Zhang et al., 2024; Zhao et al., 2025).

These modality differences also shaped the psychological profile of the mapped evidence. In the electroencephalography literature, stress, anxiety, mental fatigue, and short-latency cognitive-emotional outcomes were prominent. When interpreted through the critical-appraisal findings, however, the electroencephalography evidence should not be treated as methodologically uniform. The strongest intervention-related anxiety signal came from randomized evidence judged as having some concerns rather than low risk, whereas several acute stress, fatigue, and mood-related findings came from non-randomized studies judged serious overall or from acute paradigms in experienced practitioners. Acute studies suggested that even brief Tai Chi exposure may be associated with altered arousal- or attention-related neural indices alongside reduced perceived stress or fatigue, but those studies were often conducted in experienced practitioners or Tai Chi-trained students rather than novices, which limits their interpretability as training-onset effects (Cheung et al., 2018; Ji and Li, 2025; Wang et al., 2007). Longer electroencephalography interventions in student or older-adult samples broadened the field toward anxiety, depression, and mental-function quality of life, but the mechanistic interpretation remained variable. For example, He et al. (2024) reported psychological improvement in Tai Chi-containing arms without a clear direct neural explanation for the review-eligible psychological outcomes, whereas Wang et al. (2024) observed anxiety reductions that correlated with changes in theta oscillatory power. Accordingly, electroencephalography findings are best interpreted as suggestive evidence of state-sensitive psychophysiological change, with greater inferential weight assigned to randomized studies and substantially less causal weight assigned to serious-risk non-randomized acute studies.

The functional magnetic resonance imaging literature formed the most substantial cluster for repeated or clinically oriented studies, and it was also the modality in which the psychological outcomes appeared most clinically anchored. Studies in geriatric depression, major depressive disorder, chronic fatigue syndrome, Parkinson disease, and functional constipation used resting-state or task-based functional magnetic resonance imaging to relate Tai Chi exposure to depressive symptoms, vitality or fatigue, anxiety, emotion-regulation processes, and broader non-motor or quality-of-life constructs (Kilpatrick et al., 2022; Li X. et al., 2022; Li et al., 2024; Liu et al., 2018; Liu et al., 2020; Port et al., 2018; Teng et al., 2025; Wu et al., 2022; Xu et al., 2020; Zhang et al., 2023). Within this cluster, there was a notable recurrence of network-level interpretations involving default-mode, insular, or fronto-striatal circuitry. That recurrence should not be taken as evidence of a settled mechanistic pathway, but it does suggest that Tai Chi research in this area has moved beyond gross activation contrasts toward more network-based formulations of mind–body intervention effects. At the same time, the clinical meaning of those neural changes was not uniform across studies. Some reports aligned neural findings with depressive or fatigue-related improvement (Kilpatrick et al., 2022; Xu et al., 2020; Wu et al., 2022), whereas others found broader symptom or cognitive changes without equally clear psychological between-group differences in the main article (Li et al., 2024; Teng et al., 2025). This inconsistency indicates that neural sensitivity and psychological sensitivity may not be parallel across all populations or outcomes. Risk-of-bias judgments further qualify this interpretation. Randomized functional magnetic resonance imaging studies judged as having some concerns provide the most useful intervention-related evidence, but high-risk randomized evidence and serious-risk non-randomized or single-group studies should be interpreted as supportive rather than definitive. Cross-sectional long-term practitioner studies are valuable for identifying candidate connectivity patterns associated with Tai Chi exposure, but because exposure was self-selected, they cannot determine whether the observed network differences were caused by Tai Chi practice or reflected pre-existing participant characteristics, health behaviors, motivation, or prior exercise history.

The functional near-infrared spectroscopy literature was smaller, but it may represent one of the most strategically important directions for future work. The available studies focused on prefrontal function in relation to emotional regulation, working memory, planning, and emotional memory, with promising findings for Tai Chi-containing conditions in both acute and repeated paradigms (Li et al., 2025; Wang et al., 2025). The two additional included functional near-infrared spectroscopy studies that had not yet been fully transferred into the detailed extraction workbook at the time of drafting further suggest that this modality is beginning to expand into older-adult and clinical contexts, including depressive symptomatology during standardized Tai Chi-task performance and repeated-measures multimodal behavioral designs (Zhang et al., 2024; Zhao et al., 2025). Even so, the functional near-infrared spectroscopy evidence remains too small to support robust conclusions about modality-specific reproducibility.

### 4.2 Interpreting the concurrent neural and psychological findings

One of the specific objectives was to determine whether the literature merely reported neural and psychological outcomes in parallel or whether it explicitly tested relations between them. The added association-direction synthesis clarifies that these are distinct evidentiary situations. Several studies reported statistically significant favorable brain-behavior associations. In functional magnetic resonance imaging studies, these associations involved depressive-symptom, resilience, vitality, emotion-regulation, and cognitive-emotional outcomes. In electroencephalography/event-related-potential studies, they mainly involved anxiety or cognitive-emotional performance. In functional near-infrared spectroscopy studies, they involved emotional-memory or depression-related prefrontal activation. However, many studies contributed to the evidence map because they measured both neural and psychological outcomes, not because they tested a direct eligible association between them. This distinction is important because co-occurring neural and psychological change cannot be interpreted as evidence of neural mediation or mechanistic coupling unless a direct association, temporal sequence, mediation model, or other formal linkage analysis is reported.

Several studies did go beyond parallel reporting and examined direct brain-behavior associations or mediation models, but their inferential weight differed according to study design and risk of bias. The most informative evidence for intervention-related brain-behavior coupling came from randomized studies judged as having some concerns, whereas mediation models from cross-sectional long-term practitioner comparisons and associations from serious-risk non-randomized studies were treated as hypothesis-generating. In the functional magnetic resonance imaging literature, Kilpatrick et al. (2022) linked connectivity changes to depressive-symptom and resilience improvement, Xu et al. (2020) linked distinct right-insular connectivity changes to mood versus vitality improvement, and Liu et al. (2018, 2020) reported mediation models suggesting that connectivity patterns were statistically related to non-judgment and emotion-related outcomes in long-term practitioners. In the electroencephalography and functional near-infrared spectroscopy literature, Li Y. et al. (2022), Wang et al. (2007), Wang et al. (2024), and Wang et al. (2025) each reported neural-psychological associations that were directionally consistent with more favorable psychological or cognitive-emotional status. These studies support the plausibility of brain-behavior coupling in Tai Chi research, but the level of support is not equivalent across studies. Findings from randomized studies with some concerns provide cautious intervention-related support. Findings from cross-sectional studies support candidate association models. Findings from serious-risk non-randomized studies should be considered exploratory signals requiring replication under more rigorous designs.

However, the interpretive strength of that pattern should not be overstated. Formal association testing was not universal even among studies. Moreover, several of the available association models were secondary analyses embedded in small neuroimaging samples, in pilot studies, or in cross-sectional practitioner-control designs, where mediation language may imply more causal structure than the design can support (Kilpatrick et al., 2022; Liu et al., 2018; Liu et al., 2020). Additionally, some studies reported neural change and psychological improvement without a corresponding direct association between the two, or reported correlations only with broader symptom or physical-function indices rather than with the review-eligible psychological outcomes (He et al., 2024; Li X. et al., 2022; Teng et al., 2025). This heterogeneity matters. It suggests that the current literature is more persuasive in showing that neural and psychological changes can co-occur under Tai Chi-related conditions than in establishing a single reproducible brain-mediated pathway through which Tai Chi exerts psychological effects. The mapped evidence supports the hypothesis that Tai Chi may engage neural systems relevant to mood, fatigue, attention, emotion regulation, and cognitive-emotional processing, but it does not yet justify a generalized claim that specific neural biomarkers reliably mediate Tai Chi’s psychological effects across populations.

### 4.3 Limitations of the evidence base and of the review

The limitations of the included evidence were substantial and should directly temper interpretation. Although many randomized trials were judged as having some concerns rather than high risk, the most common issues were highly relevant to behavioral neuroscience research. These issues included incomplete reporting of allocation concealment, unavoidable lack of participant blinding in movement interventions, reliance on self-reported psychological outcomes, attrition or artifact-related loss of neural data, and limited transparency regarding prespecified analytic hierarchies for high-dimensional neural outcomes. These issues were evident in several randomized studies across electroencephalography, functional magnetic resonance imaging, and functional near-infrared spectroscopy (He et al., 2024; Ji and Li, 2025; Kilpatrick et al., 2022; Li et al., 2025; Teng et al., 2025; Wang et al., 2024). Accordingly, even the better-controlled segment of the literature remains vulnerable to selective emphasis, outcome multiplicity, and limited reproducibility. The revised synthesis therefore avoids treating all positive findings as equivalent. Studies judged high risk or serious risk were not excluded because the purpose of this scoping review was to map the full evidence landscape, but they were not used as primary support for causal or mechanistic conclusions. This distinction is important because several seemingly convergent findings came from designs vulnerable to confounding, expectancy effects, regression to the mean, incomplete neural-data retention, or self-selection into long-term Tai Chi practice.

The non-randomized and observational studies imposed additional constraints. All non-randomized intervention studies appraised with ROBINS-I were judged serious overall, primarily because of confounding and the absence of a clearly isolating Tai Chi comparator in some designs. That problem was particularly relevant in studies where all groups received Tai Chi, such that the design could illuminate change over time but not Tai Chi-specific contrast effects (Li X. et al., 2022; Wu et al., 2022). The cross-sectional studies were also intrinsically limited by self-selection. Long-term Tai Chi practitioners may differ from comparison groups in baseline motivation, health behaviors, stress regulation, or prior exercise history independently of Tai Chi itself, even when demographic matching is attempted (Li Y. et al., 2022; Liu et al., 2018; Liu et al., 2020; Port et al., 2018; Zhang et al., 2022). Those studies are valuable for identifying candidate neural and psychological signatures of long-term practice exposure, but they cannot establish causal adaptation.

The evidence base was also limited by uneven coverage across outcome domains, modalities, and settings. The evidence gap map showed clustering around cognitive-emotional or neuropsychological endpoints in electroencephalography and functional near-infrared spectroscopy studies, and around depression- and fatigue-related outcomes in functional magnetic resonance imaging studies. By contrast, stress outcomes were largely confined to electroencephalography, mental health-related quality of life and well-being outcomes were comparatively sparse outside electroencephalography and resting-state functional magnetic resonance imaging, and no included study used positron-emission tomography or magnetic resonance spectroscopy.

A further review-level limitation is that the electronic search was restricted to PubMed, Scopus, and Web of Science Core Collection. These databases provide broad biomedical, multidisciplinary, and citation-indexing coverage and were selected to capture peer-reviewed studies relevant to Tai Chi, functional brain measures, and psychological outcomes. However, relevant studies indexed only in other databases, such as Embase, PsycINFO, CINAHL, SPORTDiscus, CENTRAL, dissertations, trial registries, or other grey-literature sources, may not have been identified. Therefore, the present review should be interpreted as a systematic map of the literature retrieved through these major databases and supplementary reference-list checking, rather than as an exhaustive census of every potentially relevant report. Future reviews could extend the search to additional regional and disciplinary databases to evaluate whether the mapped evidence base changes materially.

A further limitation is that the association-direction synthesis was necessarily based on the analyses and statistics reported in the primary studies. Many studies measured neural and psychological outcomes concurrently but did not formally test eligible neural-psychological associations; others reported significant associations without complete coefficients, confidence intervals, or harmonized outcome metrics. Consequently, the added table and heatmap improve transparency about direction and consistency, but they should not be interpreted as a quantitative meta-analysis of association strength. Future studies should prespecify primary neural and psychological endpoints, report complete brain-behavior association statistics, correct for multiplicity in high-dimensional neural analyses, and align neural and psychological sampling time points to support stronger temporal and mechanistic inference.

### 4.4 Research gaps and future research priorities

The most important research gap revealed by this review is not the absence of positive findings, but the absence of a standardized architecture for testing them. Tai Chi neuroscience studies varied evidently in style, dose, instructional format, comparator choice, exposure history, neural acquisition context, and psychological outcome selection. As a result, even when multiple studies suggested broadly favorable convergence between Tai Chi practice and neural or psychological outcomes, they were often not estimating the same underlying construct in a sufficiently comparable way to permit cumulative inference. Future studies would benefit from much more standardized reporting of Tai Chi content and dose, including style or form, instructor expertise, supervised versus home-practice components, adherence, and prior participant exposure to Tai Chi or related mind–body practices.

A second priority is stronger design logic for brain-behavior questions. The field now contains enough suggestive evidence to justify trials that specify neural and psychological hypotheses *a priori* rather than treating brain-behavior associations as secondary exploratory add-ons. Adequately powered randomized studies with active comparators, repeated assessments, and explicit temporal alignment between neural and psychological measurements would be particularly valuable. For practical translation, these studies should also include pragmatic outcomes such as symptom severity, functioning, quality of life, adherence, safety, medication stability, and feasibility of implementation in rehabilitation, geriatric, mental-health, or community-care contexts. Equally important is the adoption of transparent analytic pipelines for high-dimensional neural data, including prespecified primary outcomes, correction strategies for multiple testing, and clearer justification for mediation models. Without those design features, the literature will continue to generate interesting associations without resolving whether those associations are robust, specific, or reproducible.

### 4.5 Practical implications for research and practice

For primary-study design, the mapped evidence suggests that modality selection should be driven by the specific research question rather than by convenience or disciplinary habit. Electroencephalography appears well suited to acute or state-sensitive questions involving attention, fatigue, or rapid affective shifts; functional magnetic resonance imaging appears best positioned for network-level questions involving repeated practice, clinical symptoms, and large-scale connectivity; and functional near-infrared spectroscopy may be particularly useful for prefrontal and task-based questions in paradigms that need greater compatibility with movement or ecologically realistic performance contexts (Cheung et al., 2018; Kilpatrick et al., 2022; Li et al., 2025; Wang et al., 2025). This does not imply that one modality is superior overall, but rather that the current evidence base is already differentiated enough to encourage better question-modality matching.

For clinical and behavioral application, the findings justify cautious interest rather than strong translational claims. Across several populations, Tai Chi was associated with favorable psychological or cognitive-emotional findings accompanied by neural change, and this pattern is consistent with the broader literature suggesting psychological benefits of Tai Chi in some contexts (Kraft et al., 2024; Kuang et al., 2024; Wang et al., 2010; Wang et al., 2014; Zhu et al., 2024). From a practical perspective, the mapped evidence is most relevant to contexts in which Tai Chi is considered as an adjunctive, low-intensity, non-pharmacological practice for mood, anxiety, fatigue, emotional regulation, or cognitive-emotional functioning, particularly in older adults, university students, and selected clinical populations. The findings may be especially useful for rehabilitation, geriatric, mental-health, and behavioral-medicine contexts where interventions with low physical intensity, attentional engagement, breathing regulation, and movement-based embodiment are clinically attractive. Nevertheless, this scoping review was not designed to estimate pooled effects, and the included neuroscience studies were generally too heterogeneous and methodologically limited to support definitive claims about effectiveness or mechanism. Therefore, the practical implication is not that neural measures currently validate Tai Chi as a mechanism-specific treatment, but that they provide adjunct evidence for designing more targeted clinical trials and for selecting outcomes that are sensitive to mood, fatigue, attention, and emotion-regulation change. At present, neural findings should be interpreted as informative adjunct evidence that may help refine intervention theory and trial design, rather than as validated biomarkers for routine clinical decision-making.

For practitioners, the most defensible application is to view Tai Chi as a potentially useful adjunct rather than a stand-alone replacement for established psychological, pharmacological, or rehabilitation care. The present evidence suggests that future protocols should specify the target population, symptom domain, Tai Chi dose, comparator, adherence strategy, and timing of psychological and neural assessments. In clinical populations, particularly those with depression, fatigue, Parkinson disease, functional constipation, or metabolic comorbidity, Tai Chi studies should also document usual care, medication stability, baseline functional capacity, adverse events, and feasibility outcomes. These aspects are necessary before neural findings can be translated into intervention pathways.

## 5 Conclusion

This systematic scoping review maps a growing but methodologically heterogeneous literature linking Tai Chi, brain activity, and psychological outcomes. The current evidence suggests that favorable psychological or cognitive-emotional findings often co-occur with Tai Chi-related neural changes, but direct brain-behavior analyses remain limited and uneven across modalities. Because much of the evidence is exploratory or affected by risk-of-bias concerns, these findings should be interpreted as hypothesis-generating rather than as proof of specific neural mechanisms. Future studies should use adequately powered, prospectively designed trials with prespecified neural and psychological outcomes to clarify whether Tai Chi-related brain changes mediate clinically meaningful psychological benefits.

## Data Availability

The original contributions presented in the study are included in the article/supplementary material, further inquiries can be directed to the corresponding author.

## References

[ref1] Chen Y. Wan A. Mao M. Sun W. Song Q. Mao D. (2022). Tai Chi practice enables prefrontal cortex bilateral activation and gait performance prioritization during dual-task negotiating obstacle in older adults. Front. Aging Neurosci. 14:1000427. doi: 10.3389/fnagi.2022.1000427, 36466597 PMC9716214

[ref2] Cheung T. C. Y. Liu K. P. Y. Wong J. Y. H. Bae Y.-H. Hui S. S.-C. Tsang W. W. N. . (2018). Acute effects of tai Chi training on cognitive and cardiovascular responses in late middle-aged adults: a pilot study. Evid. Based Complement. Alternat. Med. 2018:7575123. doi: 10.1155/2018/7575123, 29636784 PMC5831874

[ref3] Cui L. Yin H. Lyu S. Shen Q. Wang Y. Li X. . (2019). Tai Chi Chuan vs general aerobic exercise in brain plasticity: a multimodal MRI study. Sci. Rep. 9:17264. doi: 10.1038/s41598-019-53731-z, 31754170 PMC6872722

[ref4] He J. Chan S. H. W. Lin J. Tsang H. W. H. (2024). Integration of tai chi and repetitive transcranial magnetic stimulation for sleep disturbances in older adults: a pilot randomized controlled trial. Sleep Med. 122, 35–44. doi: 10.1016/j.sleep.2024.07.029, 39121822

[ref5] Ji R. Li J. (2025). Tai Chi Chuan teaching on alleviating mental fatigue among college students: insights from ERPs. Front. Psychol. 16:1561888. doi: 10.3389/fpsyg.2025.1561888, 40271360 PMC12015612

[ref6] Kilpatrick L. A. Siddarth P. Milillo M. M. Krause-Sorio B. Ercoli L. Narr K. L. . (2022). Impact of tai Chi as an adjunct treatment on brain connectivity in geriatric depression. J. Affect. Disord. 315, 1–6. doi: 10.1016/j.jad.2022.07.049, 35905792 PMC10182814

[ref7] Kraft J. Waibl P. J. Meissner K. (2024). Stress reduction through taiji: a systematic review and meta-analysis. BMC Complement. Med. Ther. 24:210. doi: 10.1186/s12906-024-04493-3, 38831412 PMC11149313

[ref8] Kuang X. Dong Y. Song L. Dong L. Chao G. Zhang X. . (2024). The effects of different types of tai Chi exercise on anxiety and depression in older adults: a systematic review and network meta-analysis. Front. Public Health 11:1295342. doi: 10.3389/fpubh.2023.1295342, 38259770 PMC10800705

[ref9] Larkey L. Jahnke R. Etnier J. Gonzalez J. (2009). Meditative movement as a category of exercise: implications for research. J. Phys. Act. Health 6, 230–238. doi: 10.1123/jpah.6.2.230, 19420401

[ref10] Li X. Geng J. Du X. Si H. Wang Z. (2022). Relationship between the practice of tai Chi for more than 6 months with mental health and brain in university students: an exploratory study. Front. Hum. Neurosci. 16:912276. doi: 10.3389/fnhum.2022.912276, 35814952 PMC9263293

[ref11] Li G. Huang P. Cui S. He Y. Jiang Q. Li B. . (2024). Tai Chi improves non-motor symptoms of Parkinson’s disease: one-year randomized controlled study with the investigation of mechanisms. Parkinsonism Relat. Disord. 120:105978. doi: 10.1016/j.parkreldis.2023.105978, 38244460

[ref12] Li H. Lin X. Wu X. (2025). Dual-channel mechanism of groove music fused with tai Chi to improve cognitive-emotional abilities in older adults based on a coupled fNIRS-EMG analysis: a randomized controlled study. Geroscience 47, 6583–6597. doi: 10.1007/s11357-025-01811-6, 40721570 PMC12635002

[ref13] Li Y. Wu K. Hu X. Xu T. Li Z. Zhang Y. . (2022). Altered effective connectivity of resting-state networks by tai Chi Chuan in chronic fatigue syndrome patients: a multivariate granger causality study. Front. Neurol. 13:858833. doi: 10.3389/fneur.2022.858833, 35720086 PMC9203735

[ref14] Liu Z. Li L. Liu S. Sun Y. Li S. Yi M. . (2020). Reduced feelings of regret and enhanced fronto-striatal connectivity in elders with long-term tai Chi experience. Soc. Cogn. Affect. Neurosci. 15, 861–873. doi: 10.1093/scan/nsaa111, 33007783 PMC7543941

[ref15] Liu J. Tao J. Liu W. Huang J. Xue X. Li M. . (2019). Different modulation effects of tai Chi Chuan and Baduanjin on resting-state functional connectivity of the default mode network in older adults. Soc. Cogn. Affect. Neurosci. 14, 217–224. doi: 10.1093/scan/nsz001, 30690554 PMC6374601

[ref16] Liu Z. Wu Y. Li L. Guo X. (2018). Functional connectivity within the executive control network mediates the effects of long-term tai Chi exercise on elders’ emotion regulation. Front. Aging Neurosci. 10:315. doi: 10.3389/fnagi.2018.00315, 30405392 PMC6205982

[ref17] Pan Z. Su X. Fang Q. Hou L. Lee Y. Chen C. C. . (2018). The effects of tai Chi intervention on healthy elderly by means of neuroimaging and EEG: a systematic review. Front. Aging Neurosci. 10:110. doi: 10.3389/fnagi.2018.00110, 29720936 PMC5915963

[ref18] Peters M. D. J. Marnie C. Tricco A. C. Pollock D. Munn Z. Alexander L. . (2020). Updated methodological guidance for the conduct of scoping reviews. JBI Evid. Synth. 18, 2119–2126. doi: 10.11124/JBIES-20-00167, 33038124

[ref19] Port A. P. Santaella D. F. Lacerda S. S. Speciali D. S. Balardin J. B. Lopes P. B. . (2018). Cognition and brain function in elderly tai Chi practitioners: a case-control study. EXPLORE 14, 352–356. doi: 10.1016/j.explore.2018.04.007, 30122327

[ref20] Rethlefsen M. L. Kirtley S. Waffenschmidt S. Ayala A. P. Moher D. Page M. J. . (2021). PRISMA-S: an extension to the PRISMA statement for reporting literature searches in systematic reviews. Syst. Rev. 10:39. doi: 10.1186/s13643-020-01542-z, 33499930 PMC7839230

[ref21] Sani N. A. Yusoff S. S. M. Norhayati M. N. Zainudin A. M. (2023). Tai Chi exercise for mental and physical well-being in patients with depressive symptoms: a systematic review and Meta-analysis. Int. J. Environ. Res. Public Health 20:2828. doi: 10.3390/ijerph20042828, 36833525 PMC9957102

[ref22] Solianik R. Mickevičienė D. Žlibinaitė L. Čekanauskaitė A. (2021). Tai chi improves psychoemotional state, cognition, and motor learning in older adults during the COVID-19 pandemic. Exp. Gerontol. 150:111363. doi: 10.1016/j.exger.2021.111363, 33887380 PMC8054611

[ref23] Tao J. Chen X. Liu J. Egorova N. Xue X. Liu W. . (2017). Tai Chi Chuan and Baduanjin mind-body training changes resting-state low-frequency fluctuations in the frontal lobe of older adults: a resting-state fMRI study. Front. Hum. Neurosci. 11:514. doi: 10.3389/fnhum.2017.00514, 29163096 PMC5670503

[ref24] Teng Y. Tao S. Chen J. Zhang F. Guo Y. Ying R. . (2025). Tai Chi’s synergistic modulation on autonomic nervous activity and central autonomic networks in functional constipation patients: a randomized controlled trial. Sci. Rep. 15:23560. doi: 10.1038/s41598-025-04088-z, 40603953 PMC12222652

[ref25] Tricco A. C. Lillie E. Zarin W. O’Brien K. K. Colquhoun H. Levac D. . (2018). PRISMA extension for scoping reviews (PRISMA-ScR): checklist and explanation. Ann. Intern. Med. 169, 467–473. doi: 10.7326/M18-0850, 30178033

[ref26] Wang C. Bannuru R. Ramel J. Kupelnick B. Scott T. Schmid C. H. (2010). Tai Chi on psychological well-being: systematic review and meta-analysis. BMC Complement. Altern. Med. 10:23. doi: 10.1186/1472-6882-10-23, 20492638 PMC2893078

[ref27] Wang M. Chi S. Wang X. Wang T. (2024). Effects of tai Chi on anxiety and theta oscillation power in college students during the COVID-19 pandemic: a randomized controlled trial. PLoS One 19:e0312804. doi: 10.1371/journal.pone.0312804, 39485780 PMC11530040

[ref28] Wang H. Guo Y. Fan H. Chen Z. Liu S. Zhao L. . (2025). The effects of an acute tai Chi on emotional memory and prefrontal cortex activation: a fNIRS study. Front. Behav. Neurosci. 18:1520508. doi: 10.3389/fnbeh.2024.1520508, 39911243 PMC11794301

[ref29] Wang F. Lee E.-K. O. Wu T. Benson H. Fricchione G. Wang W. . (2014). The effects of tai Chi on depression, anxiety, and psychological well-being: a systematic review and Meta-analysis. Int. J. Behav. Med. 21, 605–617. doi: 10.1007/s12529-013-9351-9, 24078491

[ref30] Wang L. Liu Y. Mimura K. Fujimoto S. (2007). The psycho-physiological effects of “Taiji sense” in Taijiquan exercise. Jpn. J. Phys. Fitness Sports Med. 56, 131–140.

[ref31] Wang M. Lyu B. (2024). Effect of 24-form simplified tai Chi on executive inhibitory control of college students: a randomized controlled trial of EEG. Front. Psychol. 15:1344989. doi: 10.3389/fpsyg.2024.1344989, 38515964 PMC10955120

[ref32] Wu K. Li Y. Zou Y. Ren Y. Wang Y. Hu X. . (2022). Tai Chi increases functional connectivity and decreases chronic fatigue syndrome: a pilot intervention study with machine learning and fMRI analysis. PLoS One 17:e0278415. doi: 10.1371/journal.pone.0278415, 36454926 PMC9714925

[ref33] Wu M.-T. Tang P.-F. Goh J. O. S. Chou T.-L. Chang Y.-K. Hsu Y.-C. . (2018). Task-switching performance improvements after tai Chi Chuan training are associated with greater prefrontal activation in older adults. Front. Aging Neurosci. 10:280. doi: 10.3389/fnagi.2018.00280, 30319391 PMC6165861

[ref34] Xu A. Zimmerman C. S. Lazar S. W. Ma Y. Kerr C. E. Yeung A. (2020). Distinct insular functional connectivity changes related to mood and fatigue improvements in major depressive disorder following tai Chi training: a pilot study. Front. Integr. Neurosci. 14:25. doi: 10.3389/fnint.2020.00025, 32581734 PMC7295154

[ref35] Yeung A. Chan J. S. M. Cheung J. C. Zou L. (2018). Qigong and tai-Chi for mood regulation. Focus (Madison). 16, 40–47. doi: 10.1176/appi.focus.20170042, 31975898 PMC6519567

[ref36] Yuan J. Zeng Q. Feng D. Wang Y. Li H. Cong Z. . (2025). The effect of 8-week tai Chi training on emotional regulation in female college students: an ERP study of N2 and P3 under a modified oddball paradigm. Front. Psychol. 16:1620704. doi: 10.3389/fpsyg.2025.1620704, 41280169 PMC12631280

[ref37] Zhang X. Bao J. Yang H. Zhang Z. Shu D. Luo L. (2022). Effects of tai Chi and Walking exercise on emotional face recognition in elderly people: an ERP study. Healthcare 10:1486. doi: 10.3390/healthcare10081486, 36011142 PMC9407806

[ref38] Zhang J. Gao T. Li Y. Song Z. Cui M. Wei Q. . (2023). The effect of Bafa Wubu of tai Chi on college students’ anxiety and depression: a randomized, controlled pilot study. Front. Physiol. 14:1036010. doi: 10.3389/fphys.2023.1036010, 36760533 PMC9905723

[ref39] Zhang J. Li Y. Liu X. Zhong D. Xue C. Fan J. . (2024). Characteristic changes of prefrontal and motor areas in patients with type 2 diabetes and major depressive disorder during a motor task of tai Chi Chuan: a functional near-infrared spectroscopy study. Brain Behav. 14:e70071. doi: 10.1002/brb3.70071, 39378277 PMC11460607

[ref40] Zhao J. Li H. Wang X. (2025). Effects of acoustically screened five-element music combined with traditional Chinese mind–body exercises on emotion regulation, working memory, and functional brain connectivity in older adults: a randomized repeated-measures study. Behav. Sci. 15:699. doi: 10.3390/bs15050699, 40426476 PMC12109339

[ref41] Zhu F. Wang Y. Yin S. Liu J. Zhong Y. Li L. (2024). The effect of tai Chi on elderly depression: a systematic review and meta-analysis of randomized controlled trials. Front. Psychol. 15. doi: 10.3389/fpsyg.2024.1489384, 39679159 PMC11637854

